# Determinants of ESG Implementation and Social Sustainability Practices in Taiwanese Hospitals: A Mixed Methods Study

**DOI:** 10.3390/healthcare14131935

**Published:** 2026-07-01

**Authors:** Yu-Hua Yan

**Affiliations:** Superintendent Office, Tainan Municipal Hospital (Managed by Show Chwan Medical Care Corporation), No. 670, Chongde Rd., East District, Tainan City 701, Taiwan; 2d0003@tmh.org.tw; Tel.: +886-6-260-9926; Fax: +886-6-260-6351

**Keywords:** environmental social and governance (ESG), healthcare sustainability, social sustainability, operational sustainability, digital healthcare, sustainability management, mixed-methods research, Taiwan

## Abstract

**Background**: Healthcare institutions increasingly face sustainability challenges associated with environmental governance, operational efficiency, digital transformation, and social responsibility in the post-pandemic era. However, limited studies have comprehensively examined the organizational factors influencing ESG implementation and healthcare social sustainability practices among hospitals. **Objective**: This study aimed to investigate the organizational determinants of ESG implementation and healthcare social sustainability practices among Taiwanese hospitals and to explore how healthcare professionals and hospital administrators perceive sustainability implementation within post-pandemic healthcare environments. **Methods**: A convergent mixed methods design integrating quantitative surveys and qualitative interviews was employed. Exploratory factor analysis (EFA), correlation analysis, and multiple regression analysis were performed, and qualitative data were analyzed using thematic analysis. **Results**: A total of 135 valid questionnaires were analyzed, and qualitative data were obtained from three semi-structured interviews. ESG sustainability support demonstrated the strongest positive influence on healthcare social sustainability practices (β = 0.481, *p* < 0.001), followed by operational sustainability (β = 0.276, *p* < 0.01) and sustainability management capability (β = 0.214, *p* < 0.05). Organizational resource pressure did not significantly influence healthcare social sustainability practices. The qualitative findings converged with and expanded upon the quantitative results by highlighting the importance of leadership support, digital healthcare transformation, operational coordination, and community health promotion in facilitating ESG implementation and long-term healthcare sustainability. **Conclusions**: The integrated findings suggest that healthcare ESG implementation increasingly functions as a comprehensive sustainability governance strategy involving operational sustainability, digital healthcare transformation, healthcare accessibility, and social responsibility practices within post-pandemic healthcare environments. Strengthening sustainability support systems, governance capability, and operational resilience may facilitate long-term healthcare social sustainability implementation.

## 1. Introduction

Healthcare institutions worldwide increasingly face sustainability challenges associated with environmental governance, operational efficiency, healthcare accessibility, workforce pressure, and organizational resilience in the post-pandemic era. In recent years, Environmental, Social, and Governance (ESG) implementation has become an increasingly important strategic priority within healthcare systems because hospitals are expected not only to provide high-quality healthcare services but also to strengthen environmental sustainability, social responsibility, and long-term organizational sustainability.

The COVID-19 pandemic further accelerated sustainability-related transformation within healthcare organizations. During the pandemic, hospitals worldwide experienced substantial operational disruption, workforce shortages, financial pressure, and healthcare accessibility challenges, thereby highlighting the importance of organizational adaptability, operational sustainability, and institutional resilience within healthcare systems [1,2]. Consequently, healthcare institutions increasingly recognize ESG implementation as part of broader healthcare sustainability management and organizational development strategies.

Previous studies have suggested that healthcare ESG implementation involves multiple organizational dimensions, including environmental sustainability, operational efficiency, digital transformation, healthcare accessibility, sustainability management capability, and social responsibility practices [3,4]. In particular, hospitals increasingly rely on sustainability-oriented management systems, digital healthcare technologies, and cross-departmental coordination mechanisms to strengthen healthcare sustainability and organizational resilience in rapidly changing healthcare environments.

Digital transformation has also become an increasingly important component of healthcare ESG implementation. Recent studies have indicated that electronic medical records, telemedicine systems, AI-assisted healthcare platforms, and digital healthcare systems may substantially improve healthcare operational efficiency, sustainability management capability, and organizational adaptability [5,6]. Furthermore, operational sustainability and resource optimization may strengthen hospitals’ long-term healthcare sustainability and service continuity under increasing healthcare pressures.

In addition to environmental sustainability and operational management, social sustainability has become another critical dimension of healthcare ESG implementation. Unlike traditional commercial organizations, hospitals operate within highly public-service-oriented environments characterized by healthcare accessibility obligations, health equity responsibilities, and strong institutional legitimacy pressures. Consequently, healthcare ESG implementation frequently involves community engagement, vulnerable population support, health promotion activities, and broader social responsibility practices [7,8].

Despite the growing importance of healthcare ESG implementation, empirical evidence regarding the determinants of ESG implementation and social sustainability practices among hospitals remains limited, particularly within Asian healthcare systems. Taiwan’s universal healthcare system and highly institutionalized healthcare environment provide a unique context for examining healthcare ESG implementation and sustainability management within hospitals. Taiwanese healthcare institutions operate under strong public-service obligations, increasing sustainability governance pressures, and extensive healthcare accessibility requirements, making Taiwan an important setting for investigating healthcare ESG implementation within post-pandemic healthcare environments.

Moreover, most previous healthcare ESG studies have primarily focused on environmental sustainability indicators or policy-oriented discussions, while relatively limited research has comprehensively examined how environmental sustainability support, operational efficiency, sustainability management capability, financial pressure, and digital transformation collectively influence hospitals’ social sustainability practices. In addition, relatively few studies have integrated quantitative statistical analysis and qualitative organizational perspectives to explore healthcare ESG implementation within real-world healthcare management contexts.

Accordingly, this study aims to identify the organizational factors associated with healthcare ESG implementation and social sustainability practices among Taiwanese hospitals and to explore healthcare professionals’ perceptions of sustainability implementation through a mixed-methods approach. This study employed a convergent mixed methods design integrating quantitative survey analysis and qualitative interviews to investigate the determinants of ESG implementation and social sustainability practices among Taiwanese hospitals. Taiwan’s universal healthcare system and highly institutionalized healthcare environment provide a unique setting for examining healthcare ESG implementation and sustainability management within hospitals. Specifically, this study examined how environmental sustainability support, operational efficiency, financial pressure, and sustainability management capability influence healthcare ESG implementation and organizational sustainability practices within post-pandemic healthcare environments.

## 2. Literature Review

### 2.1. Healthcare ESG Implementation and Sustainability Management

Environmental, Social, and Governance (ESG) implementation has become an increasingly important strategic issue within healthcare systems in recent years. Originally developed within the corporate sustainability and investment sectors, ESG frameworks are now widely applied across healthcare organizations because hospitals increasingly face sustainability pressures associated with environmental governance, operational efficiency, healthcare accessibility, workforce sustainability, and social responsibility [3].

Within healthcare environments, ESG implementation extends beyond traditional environmental management activities and increasingly involves broader organizational sustainability practices. Previous studies have suggested that healthcare ESG includes environmental sustainability initiatives, operational sustainability, healthcare accessibility, digital transformation, organizational resilience, community engagement, and social responsibility practices [3,4].

Environmental sustainability has become one of the most visible dimensions of healthcare ESG implementation. Hospitals are major consumers of energy, water, medical materials, and healthcare resources and therefore generate substantial environmental impact through carbon emissions, medical waste, and resource consumption [9]. Consequently, many healthcare institutions have increasingly implemented carbon reduction initiatives, green procurement systems, energy conservation strategies, and environmentally sustainable operational practices.

However, previous studies have suggested that healthcare ESG implementation should not be understood solely as environmental management. Rather, ESG within healthcare systems increasingly functions as a multidimensional sustainability framework integrating organizational governance, operational efficiency, healthcare accessibility, and social sustainability [3]. This broader interpretation became particularly important during the COVID-19 pandemic because healthcare organizations were required to simultaneously maintain healthcare accessibility, operational resilience, workforce sustainability, and public-service responsibilities under rapidly changing healthcare conditions.

In addition, healthcare ESG implementation differs substantially from ESG implementation within commercial industries. Unlike profit-oriented corporations, hospitals operate within highly public-service-oriented institutional environments characterized by strong healthcare accessibility obligations, health equity expectations, and institutional legitimacy pressures [7]. Consequently, healthcare organizations frequently regard sustainability implementation as part of their institutional missions and public-service responsibilities rather than solely as financial or reputational strategies.

Previous studies have also emphasized that healthcare ESG implementation may strengthen long-term organizational resilience and sustainability capability. He and Harris [1] argued that the COVID-19 pandemic accelerated organizational transformation and reinforced the importance of sustainability-oriented management systems within healthcare organizations. Similarly, Heggen et al. (2020) [2] suggested that healthcare sustainability governance increasingly requires organizational adaptability, ethical governance systems, and long-term institutional resilience.

**Figure 1:**
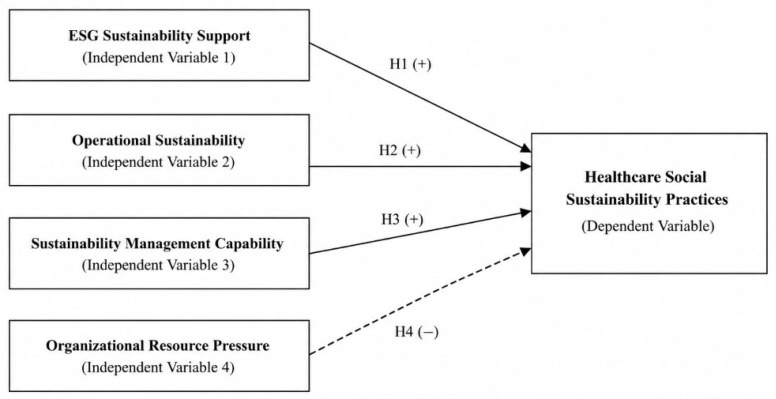
Proposed conceptual framework of the determinants of healthcare social sustainability practices in Taiwanese hospitals. Note: Solid lines indicate hypothesized positive relationships; dashed line indicates hypothesized negative relationship.

Despite the growing importance of healthcare ESG implementation, empirical evidence regarding the determinants of ESG implementation and social sustainability practices within hospitals remains relatively limited, particularly within Asian healthcare systems. Moreover, relatively few studies have comprehensively examined how operational sustainability, strategic sustainability management capability, digital transformation, and institutional support collectively influence healthcare ESG implementation within post-pandemic healthcare environments. Based on the literature review and theoretical foundations, a conceptual framework was developed to examine the relationships among ESG sustainability support, operational sustainability, organizational resource pressure, sustainability management capability, and healthcare social sustainability practices. The proposed framework is presented in Figure 1.

### 2.2. Operational Sustainability and Digital Transformation in Healthcare Organizations

Operational sustainability has become an increasingly important component of healthcare ESG implementation. Hospitals worldwide continue facing increasing healthcare demand, workforce shortages, rising operational costs, and financial pressures, thereby making operational efficiency and resource optimization critical aspects of long-term healthcare sustainability management.

Previous studies have suggested that operational sustainability may substantially influence healthcare organizations’ ability to maintain healthcare quality, organizational adaptability, and social sustainability practices under resource-constrained environments [8]. Effective operational sustainability management may improve hospitals’ capacity to allocate healthcare resources efficiently, optimize healthcare workflows, and maintain long-term organizational resilience.

Digital transformation has also become increasingly integrated into healthcare sustainability governance and ESG implementation. Recent advances in healthcare information technology, telemedicine systems, electronic medical records, AI-assisted healthcare platforms, and digital healthcare systems have significantly transformed healthcare management and operational processes [5].

Digital healthcare systems may improve healthcare accessibility, strengthen governance coordination, reduce operational inefficiencies, and enhance organizational adaptability within rapidly changing healthcare environments [10]. During the COVID-19 pandemic, digital healthcare technologies became particularly important because telemedicine systems and digital healthcare services enabled healthcare institutions to maintain healthcare continuity and organizational flexibility under substantial healthcare system pressure.

Previous studies have further emphasized that operational sustainability and digital transformation may interact closely with healthcare ESG implementation. Hospitals possessing stronger digital capability and operational efficiency may demonstrate greater organizational capacity to sustain long-term environmental governance and social sustainability practices. Consequently, operational sustainability and digital transformation increasingly function as important determinants of healthcare ESG implementation within healthcare organizations.

Nevertheless, empirical studies examining how operational sustainability and digital transformation influence healthcare ESG implementation remain relatively limited. In particular, limited research has investigated how digital transformation capability and operational efficiency jointly contribute to hospitals’ social sustainability practices within post-pandemic healthcare environments.

### 2.3. Sustainability Management Capability and Organizational Sustainability

Sustainability management capability represents another important determinant of healthcare ESG implementation. Sustainability management capability generally refers to hospitals’ ability to coordinate organizational resources, integrate sustainability strategies, facilitate cross-departmental collaboration, and implement long-term organizational transformation effectively.

The Resource-Based View (RBV) suggests that organizations achieve sustainable advantages through valuable organizational resources and strategic capabilities [11,12]. Within healthcare organizations, strategic resources may include leadership systems, governance coordination capability, digital transformation competence, sustainability-oriented organizational culture, and institutional trust.

Previous studies have suggested that sustainability management capability and sustainability-oriented management systems substantially influence hospitals’ sustainability implementation and organizational resilience [3,8].

Leadership commitment also plays an important role in healthcare ESG implementation. Previous organizational governance studies have emphasized that executive leadership support may substantially influence sustainability-oriented organizational culture, governance integration, and long-term sustainability implementation effectiveness [13,14].

In addition, organizational readiness for change may influence hospitals’ ESG transformation processes. Weiner [15] suggested that organizational readiness reflects collective organizational commitment and shared capability to implement complex organizational change initiatives. Within healthcare systems, ESG implementation may therefore depend not only on sustainability policies but also on leadership engagement, organizational coordination systems, and institutional adaptability.

Moreover, sustainability-oriented organizational culture may strengthen healthcare ESG implementation by improving staff engagement, interdepartmental collaboration, and organizational sustainability awareness. Hall [16] further suggested that intangible organizational resources, including institutional culture and governance systems, may substantially influence long-term organizational sustainability and strategic performance.

Despite the recognized importance of sustainability management capability, relatively limited empirical studies have comprehensively examined how strategic sustainability management capability influences healthcare ESG implementation and social sustainability practices among hospitals, particularly within Asian healthcare environments.

### 2.4. Social Sustainability Practices in Healthcare Systems

Social sustainability has become an increasingly important dimension of healthcare ESG implementation. Unlike traditional environmental sustainability initiatives, social sustainability within healthcare systems primarily focuses on healthcare accessibility, health equity, community engagement, workforce sustainability, vulnerable population support, and broader public-service responsibilities.

Previous studies have suggested that healthcare organizations possess substantial social responsibility obligations because hospitals directly influence public health outcomes, healthcare accessibility, and community well-being [7]. Consequently, healthcare ESG implementation frequently includes health promotion activities, community outreach programs, healthcare accessibility initiatives, and support for vulnerable populations.

The COVID-19 pandemic further reinforced the importance of social sustainability within healthcare systems. During the pandemic, healthcare institutions were required to maintain healthcare accessibility, support vulnerable populations, and sustain healthcare workforce stability under substantial operational and financial pressure [1].

Previous studies have also suggested that social sustainability practices may strengthen institutional legitimacy and organizational trust within healthcare environments. Healthcare organizations demonstrating stronger social sustainability commitment may improve public trust, organizational reputation, and stakeholder engagement [8].

However, maintaining long-term social sustainability practices frequently requires substantial institutional support, operational sustainability, governance coordination, and organizational adaptability. Hospitals experiencing workforce shortages, financial pressure, and operational instability may face greater difficulty sustaining long-term social responsibility initiatives and healthcare accessibility programs.

Despite the increasing importance of social sustainability within healthcare ESG implementation, relatively limited studies have comprehensively examined the factors influencing hospitals’ social sustainability practices, particularly within post-pandemic healthcare environments. Furthermore, few studies have integrated quantitative and qualitative perspectives to explore how healthcare organizations operationalize social sustainability within real-world healthcare management contexts.

### 2.5. Research Questions

Based on the existing healthcare sustainability literature and the identified research gaps, this study addressed the following research questions:

RQ1: What factors influence ESG implementation and social sustainability practices among Taiwanese hospitals?
RQ2: How do environmental sustainability support, operational efficiency, financial pressure, and strategic sustainability management capability affect healthcare ESG implementation?
RQ3: How do healthcare professionals and hospital administrators perceive ESG implementation and sustainability practices within post-pandemic healthcare environments?
RQ4: How does digital transformation contribute to healthcare sustainability and ESG implementation within Taiwanese hospitals?

## 3. Methodology

### 3.1. Research Design

This study employed a mixed methods approach integrating quantitative survey analysis and qualitative interviews to investigate factors associated with ESG implementation and healthcare social sustainability practices among Taiwanese hospitals in the post-pandemic era. Mixed methods research enables the integration of quantitative statistical analysis and qualitative contextual interpretation to provide more comprehensive understanding regarding complex healthcare sustainability phenomena [17].

Because healthcare ESG implementation involves environmental sustainability support, operational sustainability, digital healthcare management, organizational resource conditions, and healthcare social responsibility practices, a mixed methods approach was considered appropriate for examining both measurable organizational relationships and practical healthcare sustainability experiences within real-world healthcare settings.

The quantitative component examined the relationships among ESG sustainability support, operational sustainability, organizational resource pressure, sustainability management capability, and healthcare social sustainability practices. The qualitative component explored how healthcare professionals and hospital administrators perceive ESG implementation and sustainability management within post-pandemic healthcare environments.

Quantitative and qualitative data were collected during overlapping periods and integrated during the interpretation stage. This approach enabled simultaneous examination of measurable sustainability implementation factors and contextual healthcare sustainability experiences associated with ESG implementation.

### 3.2. Study Setting and Participants

#### 3.2.1. Quantitative Survey

The quantitative component employed convenience sampling to recruit healthcare professionals and hospital management personnel from Taiwanese healthcare institutions. Participants included hospital administrators, department managers, healthcare professionals, ESG-related personnel, and individuals involved in hospital sustainability management activities.

Questionnaire data were collected between January 2025 and April 2025. Eligible participants were required to be employed in Taiwanese healthcare institutions and have experience related to healthcare administration, sustainability management, ESG implementation, or hospital operations.

Inclusion criteria were the following: (1) employment in a Taiwanese healthcare institution and (2) involvement in healthcare administration, sustainability management, ESG implementation, or hospital operational activities. Questionnaires were considered invalid if more than 20% of items were missing, if response patterns indicated obvious non-engagement (e.g., identical responses across all items), or if substantial sections of the questionnaire were incomplete.

#### 3.2.2. Qualitative Interviews

The qualitative component utilized purposive sampling to recruit senior hospital administrators and ESG-related management personnel with substantial experience in healthcare sustainability implementation and hospital operational management.

Semi-structured interviews were conducted to obtain in-depth perspectives regarding ESG implementation, sustainability governance, digital healthcare transformation, organizational support systems, and healthcare social sustainability practices.

### 3.3. Instrument Development

The questionnaire instrument was developed based on previous healthcare sustainability and ESG implementation literature [3,4]. The survey instrument focused on hospitals’ sustainability support systems, operational sustainability, organizational resource conditions, sustainability management capability, and healthcare social sustainability practices.

The questionnaire consisted of five major dimensions:

1. ESG sustainability support.
2. Operational sustainability.
3. Organizational resource pressure.
4. Sustainability management capability.
5. Healthcare social sustainability practices.

Most questionnaire items were measured using a five-point Likert scale ranging from 1 (“strongly disagree”) to 5 (“strongly agree”).

The qualitative interview guide was developed based on the same conceptual framework and focused on healthcare ESG implementation experiences, digital healthcare sustainability, operational sustainability, healthcare management challenges, and healthcare social responsibility practices.

Prior to formal data collection, the questionnaire content was reviewed by healthcare management experts and hospital administrators to ensure content validity and wording clarity. The questionnaire items used in this study are provided in Supplementary File S1.

### 3.4. Data Collection Procedures

The quantitative questionnaire survey was distributed electronically and in paper format to eligible participants from Taiwanese healthcare institutions between January 2025 and April 2025. Prior to participation, respondents were informed regarding the study purpose, confidentiality protection, anonymity, and voluntary participation principles.

For the qualitative component, semi-structured interviews were conducted between March 2025 and May 2025 through face-to-face meetings or online communication platforms depending on participant availability and institutional arrangements.

The interview questions focused on ESG implementation experiences, sustainability management practices, digital healthcare transformation, operational sustainability, organizational coordination processes, and healthcare social sustainability activities within post-pandemic healthcare environments.

Each interview lasted approximately 40–90 min and was audio-recorded with participant consent. The interview recordings were subsequently transcribed verbatim for thematic analysis.

To strengthen interpretive rigor and methodological triangulation, quantitative statistical findings and qualitative thematic findings were integrated during the interpretation stage.

### 3.5. Measures

#### 3.5.1. ESG Sustainability Support

This construct evaluated hospitals’ ESG sustainability support systems, environmental sustainability initiatives, healthcare sustainability implementation mechanisms, and organizational sustainability support structures.

#### 3.5.2. Operational Sustainability

This construct measured hospitals’ operational efficiency, workflow sustainability, resource optimization capability, and healthcare operational management effectiveness.

#### 3.5.3. Organizational Resource Pressure

This construct assessed perceived financial burden, workforce pressure, operational constraints, and resource-related organizational challenges experienced within healthcare institutions.

#### 3.5.4. Sustainability Management Capability

This construct evaluated hospitals’ sustainability management capability, including leadership support, interdepartmental coordination, sustainability implementation systems, and organizational support mechanisms associated with ESG implementation.

#### 3.5.5. Healthcare Social Sustainability Practices

This construct assessed hospitals’ healthcare social sustainability practices, including community engagement, healthcare accessibility initiatives, health promotion activities, and vulnerable population support programs.

### 3.6. Data Analysis

#### 3.6.1. Quantitative Analysis

Quantitative data were analyzed using descriptive statistics, reliability analysis, exploratory factor analysis, correlation analysis, and multiple regression analysis.

Exploratory Factor Analysis (EFA) with Varimax rotation was performed to examine the underlying factor structure of the ESG-related constructs. Kaiser–Meyer–Olkin (KMO) and Bartlett’s Test of Sphericity were conducted to evaluate sampling adequacy and factorability.

Pearson correlation analysis was conducted to examine relationships among the study variables. Multiple regression analysis was further performed to investigate the influence of ESG sustainability support, operational sustainability, organizational resource pressure, and sustainability management capability on hospitals’ healthcare social sustainability practices.

#### 3.6.2. Qualitative Analysis

The qualitative interview data were analyzed using thematic analysis following the procedures proposed by Braun and Clarke [18]. First, the interview transcripts were repeatedly reviewed to achieve data familiarization. Second, initial codes were generated to identify meaningful ESG implementation and healthcare sustainability concepts. Third, similar codes were grouped into broader thematic categories reflecting healthcare sustainability implementation, digital healthcare sustainability, operational sustainability, organizational challenges, and healthcare social sustainability practices.

The qualitative findings were subsequently integrated with quantitative statistical findings during the interpretation stage to strengthen contextual understanding and methodological triangulation.

### 3.7. Reliability and Validity

Cronbach’s alpha coefficients were used to evaluate internal consistency reliability for all study constructs. The questionnaire demonstrated satisfactory reliability levels across all dimensions.

Construct validity was evaluated using Exploratory Factor Analysis (EFA). Kaiser–Meyer–Olkin (KMO) and Bartlett’s Test of Sphericity further confirmed the suitability of the data for factor analysis.

For the qualitative component, research credibility was strengthened through repeated transcript review, thematic consistency examination, and triangulation between quantitative statistical findings and qualitative thematic findings.

### 3.8. Ethical Considerations

This study was approved by the Institutional Review Board of Tainan Municipal Hospital (IRB No. 1131001 on 5 November 2024). All procedures were conducted in accordance with relevant ethical guidelines and institutional regulations.

Prior to participation, all participants were informed about the purpose of the study, confidentiality protection, anonymity, and voluntary participation principles. Written informed consent was obtained from all interview participants before the interviews and audio recordings were conducted.

The requirement for written informed consent for the questionnaire survey was waived because the survey was anonymous and posed minimal risk to participants.

All questionnaire responses, interview recordings, and transcript data were anonymized and used solely for academic research purposes.

## 4. Results

### 4.1. Participant Characteristics

A total of 138 questionnaires were collected during the study period. After excluding incomplete and invalid responses, 135 valid questionnaires were included in the final statistical analysis. The participants included healthcare professionals, hospital administrators, department managers, ESG-related personnel, and individuals involved in healthcare sustainability implementation within Taiwanese healthcare institutions. The respondents represented diverse institutional and professional backgrounds, thereby providing broad perspectives regarding ESG implementation and healthcare sustainability practices within post-pandemic healthcare environments.

As shown in Table 1, female participants accounted for the majority of respondents (68.1%). The largest age group was 41–50 years (34.1%), followed by 31–40 years (28.9%). More than half of the participants were employed in regional hospitals (54.8%), while 20.7% worked in medical centers and 24.5% worked in district hospitals. Regarding professional roles, healthcare professionals represented the largest proportion of respondents (60.0%), followed by administrative managers (21.5%) and ESG/sustainability-related personnel (18.5%). In addition, 65.2% of participants reported having prior ESG-related experience, indicating that most respondents possessed familiarity with healthcare sustainability implementation and ESG-related management practices (Table 1).

For the qualitative component, three semi-structured interviews were conducted with senior hospital administrators and ESG-related management personnel between March 2025 and May 2025. The interview participants possessed substantial experience in healthcare sustainability implementation, hospital operations, and healthcare management coordination.

Three senior hospital administrators and ESG-related management personnel participated in the qualitative interviews. Their characteristics are summarized in Table 2.

### 4.2. Reliability and Validity Analysis

To evaluate the reliability and construct validity of the questionnaire instrument, Cronbach’s alpha coefficients, Kaiser–Meyer–Olkin (KMO) measure, and Bartlett’s Test of Sphericity were conducted prior to factor analysis.

The results demonstrated satisfactory internal consistency across all study constructs. Cronbach’s alpha values ranged from 0.826 to 0.961, indicating good to excellent reliability levels. ESG sustainability support (α = 0.957), operational sustainability (α = 0.959), sustainability management capability (α = 0.961), and healthcare social sustainability practices (α = 0.959) demonstrated particularly high internal consistency (Table 3).

The Kaiser–Meyer–Olkin (KMO) value was 0.875, indicating adequate sampling suitability for factor analysis. Bartlett’s Test of Sphericity was statistically significant, χ^2^ (1485) = 8870.450, *p* < 0.001, suggesting that the correlation matrix was appropriate for exploratory factor analysis (Table 4).

Overall, these findings indicate that the measurement instrument demonstrated satisfactory reliability and construct validity for subsequent statistical analyses.

### 4.3. Exploratory Factor Analysis

Exploratory Factor Analysis (EFA) was conducted to examine the underlying factor structure of the ESG-related constructs and identify the major dimensions associated with healthcare ESG implementation and sustainability practices.

Principal component analysis with Varimax rotation was employed for factor extraction. Factors with eigenvalues greater than 1.0 were retained according to Kaiser’s criterion. The results identified four major factors associated with healthcare ESG implementation.

The extracted factors included the following:

1. ESG Sustainability Support;
2. Operational Sustainability;
3. Sustainability Management Capability;
4. Healthcare Social Sustainability Practices.

These four factors collectively explained 73.31% of the total variance, indicating satisfactory construct validity and factor structure stability (Table 5).

The first factor reflected hospitals’ ESG sustainability support systems, environmental sustainability initiatives, and healthcare sustainability implementation mechanisms. The second factor primarily represented operational efficiency, workflow sustainability, resource optimization capability, and digital healthcare management processes.

The third factor included leadership support, sustainability implementation systems, organizational support mechanisms, and sustainability management coordination capability. The fourth factor reflected hospitals’ healthcare social sustainability practices, including community engagement, healthcare accessibility initiatives, health promotion activities, and support for vulnerable populations.

Items with factor loadings below 0.50 or substantial cross-loadings were excluded to improve factor clarity and construct interpretability. Although the questionnaire was initially developed based on five conceptual dimensions derived from the literature, exploratory factor analysis indicated that several conceptually related items clustered together empirically. The final four-factor structure reflected the underlying response patterns observed in the study sample while remaining broadly consistent with the proposed theoretical framework.

### 4.4. Correlation Analysis

Pearson correlation analysis was conducted to examine the relationships among ESG sustainability support, operational sustainability, organizational resource pressure, sustainability management capability, and healthcare social sustainability practices.

ESG sustainability support demonstrated significant positive correlations with healthcare social sustainability practices (r = 0.742, *p* < 0.001). Operational sustainability also showed significant positive correlations with healthcare social sustainability practices (r = 0.658, *p* < 0.001).

Sustainability management capability demonstrated strong positive correlations with ESG sustainability support (r = 0.721, *p* < 0.001) and healthcare social sustainability practices (r = 0.694, *p* < 0.001). Organizational resource pressure demonstrated relatively weak negative correlations with the other study variables (Table 6).

Overall, the findings indicate that hospitals possessing stronger ESG sustainability support systems, operational sustainability capability, and sustainability management capability tended to demonstrate higher levels of healthcare social sustainability implementation.

### 4.5. Multiple Regression Analysis

Multiple regression analysis was conducted to examine the influence of ESG sustainability support, operational sustainability, organizational resource pressure, and sustainability management capability on hospitals’ healthcare social sustainability practices.

Prior to regression analysis, multicollinearity diagnostics were performed. The variance inflation factor (VIF) values ranged from 1.214 to 2.487, indicating that multicollinearity was not a significant concern. The Durbin–Watson statistic was 1.964, suggesting no substantial autocorrelation problem.

The overall regression model was statistically significant (F = 42.318, *p* < 0.001) and explained 68.4% of the variance in healthcare social sustainability practices (Adjusted R^2^ = 0.684).

ESG sustainability support demonstrated the strongest positive influence on healthcare social sustainability practices (β = 0.481, *p* < 0.001), followed by operational sustainability (β = 0.276, *p* < 0.01) and sustainability management capability (β = 0.214, *p* < 0.05). Organizational resource pressure did not demonstrate a statistically significant negative effect (Table 7).

These findings suggest that hospitals possessing stronger ESG sustainability support systems, operational sustainability capability, and sustainability management capability may demonstrate greater capacity to maintain long-term healthcare social sustainability practices within post-pandemic healthcare environments.

### 4.6. Qualitative Findings

The qualitative analysis identified four major themes associated with healthcare ESG implementation and organizational sustainability transformation within Taiwanese hospitals: (1) integration of ESG implementation into hospital operational systems, (2) organizational support and leadership engagement, (3) digital healthcare transformation and operational adaptability, and (4) community health promotion and healthcare accessibility.

Participants consistently emphasized that ESG implementation within healthcare institutions has gradually evolved beyond traditional environmental management activities and increasingly functions as an integrated organizational sustainability strategy embedded within hospital governance systems, operational coordination, and long-term institutional planning processes.

One participant explained:

*“ESG is no longer only about environmental protection. It has become part of our hospital sustainability management and operational planning.”*

Leadership engagement and organizational support were also repeatedly identified as important determinants of successful ESG implementation. Participants emphasized that executive support and cross-departmental coordination substantially influence whether sustainability-oriented practices become integrated into routine hospital operations and organizational decision-making processes.

One participant noted:

*“Leadership support significantly influences whether ESG implementation becomes embedded into daily hospital operations.”*

Digital healthcare transformation further emerged as an important facilitator of healthcare ESG implementation and organizational adaptability. Participants frequently described electronic medical records, AI-assisted healthcare platforms, telemedicine systems, and digital governance systems as important organizational tools improving operational efficiency, communication coordination, and sustainability management capability within hospitals.

For example, one participant stated:

*“Digital systems and AI-assisted platforms have significantly improved operational efficiency and sustainability management.”*

Participants also emphasized that community health promotion and healthcare accessibility remain important organizational responsibilities despite workforce shortages and increasing operational pressure. Several participants indicated that hospitals continue implementing community-oriented healthcare services and accessibility initiatives because these activities are regarded as essential components of healthcare social responsibility and long-term organizational mission.

One participant explained:

*“Community health promotion remains an important part of our hospital mission even under operational pressure.”*

Overall, the qualitative findings identified four major themes related to governance coordination, operational sustainability, digital healthcare transformation, organizational support mechanisms, and healthcare social sustainability practices. Rather than functioning solely as environmental management activities, ESG implementation increasingly appears to operate as a broader organizational sustainability governance framework within post-pandemic healthcare environments.

## 5. Discussion

The qualitative findings largely confirmed and expanded the quantitative results, providing contextual explanations for the significant effects of ESG sustainability support, operational sustainability, and sustainability management capability on healthcare social sustainability practices.

### 5.1. Integration of Quantitative and Qualitative Findings

This study employed a convergent mixed methods design to investigate the determinants of ESG implementation and healthcare social sustainability practices among Taiwanese hospitals. The quantitative findings demonstrated that ESG sustainability support, operational sustainability, and sustainability management capability significantly influenced healthcare social sustainability practices, whereas organizational resource pressure did not exert a statistically significant effect.

The qualitative findings largely converged with and expanded upon these quantitative results. Participants consistently emphasized that ESG implementation has gradually evolved beyond traditional environmental management activities and increasingly functions as an integrated organizational sustainability strategy embedded within hospital governance systems, operational coordination processes, and long-term institutional planning. In particular, leadership engagement, digital healthcare transformation, and cross-departmental collaboration were repeatedly identified as important facilitators of ESG implementation.

The integration of quantitative and qualitative findings provides a more comprehensive understanding of healthcare ESG implementation within Taiwanese hospitals. While the quantitative analysis identified the relative importance of key organizational determinants, the qualitative findings explained how these factors operate within real-world healthcare environments. For example, ESG sustainability support emerged as the strongest statistical predictor of healthcare social sustainability practices, and interview participants further described how sustainability support systems facilitate organizational coordination, sustainability planning, and long-term implementation efforts.

Similarly, operational sustainability and sustainability management capability demonstrated significant positive relationships with healthcare social sustainability practices in the quantitative analysis. The qualitative findings further revealed that digital healthcare systems, leadership support, and governance coordination mechanisms strengthen hospitals’ ability to maintain sustainability initiatives under increasingly complex healthcare conditions.

Overall, the convergence between quantitative and qualitative evidence suggests that healthcare ESG implementation increasingly functions as a comprehensive sustainability governance framework rather than a collection of isolated environmental activities. This integrated interpretation strengthens the validity of the study findings and provides a richer understanding of healthcare sustainability implementation within post-pandemic healthcare environments.

### 5.2. ESG Sustainability Support and Social Sustainability

Among all explanatory variables, ESG sustainability support demonstrated the strongest positive influence on healthcare social sustainability practices. The quantitative findings indicate that hospitals possessing stronger sustainability support systems are more likely to implement healthcare social sustainability initiatives, including community health promotion, healthcare accessibility enhancement, and socially responsible healthcare practices.

The qualitative findings further supported this relationship. Participants consistently emphasized that ESG implementation has become increasingly integrated into organizational planning, sustainability governance mechanisms, and long-term institutional development strategies. Several interviewees indicated that sustainability support systems facilitate organizational coordination and strengthen hospitals’ commitment to social responsibility activities beyond routine healthcare service delivery.

These findings are consistent with previous studies suggesting that healthcare sustainability requires comprehensive organizational support systems integrating environmental responsibility, social commitment, and governance coordination [7,9]. Healthcare institutions increasingly recognize that sustainability implementation extends beyond environmental management activities and contributes to broader organizational sustainability and social value creation. In addition, the findings support the view that sustainability-oriented organizational environments may strengthen hospitals’ capacity to maintain long-term healthcare accessibility and community engagement initiatives [10].

The integration of quantitative and qualitative findings suggests that ESG sustainability support functions not only as a structural resource but also as an organizational mechanism that promotes sustainability-oriented decision-making and institutional commitment. Consequently, sustainability support systems appear to play a central role in facilitating healthcare social sustainability practices within post-pandemic healthcare environments.

### 5.3. Operational Sustainability, Digital Transformation, and ESG Implementation

The quantitative analysis demonstrated that operational sustainability significantly influenced healthcare social sustainability practices. Hospitals with stronger operational sustainability capacity were more likely to report higher levels of healthcare social sustainability implementation.

The qualitative findings provided important contextual explanations for this relationship. Participants frequently identified digital healthcare systems, electronic medical records, telemedicine services, AI-assisted healthcare platforms, and data-driven management systems as important contributors to operational efficiency and organizational adaptability. Interviewees emphasized that digital healthcare technologies have become increasingly important for maintaining healthcare service continuity and sustainability under rapidly changing healthcare conditions.

These findings are consistent with previous studies suggesting that digital transformation may improve organizational adaptability, operational coordination, and long-term sustainability performance within healthcare organizations [5,10]. Digital healthcare systems may facilitate resource allocation, communication efficiency, and service accessibility while supporting sustainability-oriented operational processes. These findings are consistent with previous studies suggesting that digital transformation may improve organizational adaptability, operational coordination, and long-term sustainability performance within healthcare organizations [5,10]. Recent research has further proposed that hospital sustainability should be evaluated through an integrated framework combining ESG and digital transformation, highlighting digital capability as a key component of long-term organizational sustainability [19]. Digital healthcare systems may facilitate resource allocation, communication efficiency, and service accessibility while supporting sustainability-oriented operational processes.

The qualitative findings further revealed that operational sustainability and digital transformation are closely interconnected. Participants perceived digital healthcare technologies as practical tools supporting operational resilience and healthcare accessibility. Therefore, operational sustainability appears to function not only through resource optimization but also through hospitals’ ability to integrate digital innovations into sustainability-oriented healthcare management practices.

Overall, the findings suggest that operational sustainability and digital transformation jointly contribute to healthcare ESG implementation and long-term healthcare social sustainability practices.

### 5.4. Sustainability Management Capability and Organizational Readiness

The quantitative findings indicated that sustainability management capability significantly influenced healthcare social sustainability practices. Hospitals possessing stronger sustainability management capability were more likely to implement sustainability-oriented organizational practices and healthcare social responsibility initiatives.

The qualitative findings reinforced this relationship by highlighting the importance of leadership engagement, governance coordination, and organizational commitment in facilitating ESG implementation. Participants repeatedly emphasized that successful sustainability implementation requires executive support, cross-departmental collaboration, and clearly defined sustainability objectives.

These findings are consistent with organizational readiness theory, which suggests that organizational capability and collective commitment are important determinants of successful organizational change and implementation processes [15]. Sustainability implementation within healthcare organizations often requires substantial coordination among multiple professional groups and organizational units. Consequently, hospitals with stronger governance capability and organizational readiness may be better positioned to integrate sustainability initiatives into routine organizational activities.

The integration of quantitative and qualitative evidence suggests that sustainability management capability represents both a strategic and operational resource. Beyond formal sustainability policies, hospitals require leadership support, organizational readiness, and governance mechanisms that facilitate sustainability-oriented decision-making and implementation. Therefore, sustainability management capability appears to be a critical determinant of long-term ESG implementation within healthcare institutions. The integration of quantitative and qualitative evidence suggests that sustainability management capability represents both a strategic and operational resource. Beyond formal sustainability policies, hospitals require leadership support, organizational readiness, and governance mechanisms that facilitate sustainability-oriented decision-making and implementation. This interpretation is consistent with recent evidence indicating that sustainability management control systems have become an essential governance mechanism supporting strategic decision-making and long-term sustainability implementation in healthcare organization [20].

### 5.5. Organizational Resource Pressure and Institutional Commitment

Contrary to expectations, organizational resource pressure did not demonstrate a statistically significant influence on healthcare social sustainability practices. Although the relationship was negative, the effect did not reach statistical significance.

The qualitative findings provide a potential explanation for this result. Participants acknowledged that workforce shortages, increasing healthcare demand, and operational pressures continue to challenge healthcare organizations. Nevertheless, many interviewees emphasized that hospitals remain committed to community health promotion, healthcare accessibility, and social responsibility activities despite these constraints.

This finding suggests that institutional commitment to healthcare social sustainability may persist even under conditions of resource limitation. Participants frequently described sustainability-related activities as essential components of hospital missions rather than discretionary organizational initiatives. Consequently, hospitals may continue supporting social sustainability practices despite experiencing operational and financial pressures.

The findings also align with institutional theory, which suggests that organizations often maintain socially valued practices to preserve legitimacy and stakeholder trust despite environmental constraints [12,13]. Therefore, organizational resource pressure alone may not be sufficient to explain variations in healthcare social sustainability practices within healthcare institutions.

Overall, the findings indicate that healthcare social sustainability practices may be influenced more strongly by organizational commitment, governance support, and sustainability capability than by resource pressure alone.

### 5.6. Practical Implications

The findings provide several practical implications for healthcare administrators and policymakers. First, healthcare organizations should strengthen ESG sustainability support systems because sustainability support demonstrated the strongest influence on healthcare social sustainability practices. Hospitals may benefit from integrating sustainability objectives into organizational governance structures, strategic planning processes, and performance management systems.

Second, healthcare institutions should continue investing in operational sustainability and digital healthcare transformation. The qualitative findings highlighted the important role of digital healthcare systems in improving operational efficiency, organizational adaptability, and healthcare accessibility. Digital transformation initiatives may therefore contribute to both healthcare sustainability and service quality improvement.

Third, leadership engagement and sustainability management capability should be strengthened to facilitate long-term ESG implementation. Organizational readiness, governance coordination, and executive commitment appear essential for embedding sustainability initiatives into routine healthcare operations.

Finally, policymakers may consider developing supportive frameworks that encourage hospitals to integrate sustainability objectives into healthcare management practices while maintaining healthcare accessibility and community engagement.

### 5.7. Limitations and Future Research

Several limitations should be acknowledged. First, the study employed a cross-sectional design, which limits causal inference regarding the relationships among ESG sustainability support, operational sustainability, sustainability management capability, and healthcare social sustainability practices.

Second, the study focused on Taiwanese hospitals; therefore, the generalizability of the findings to other healthcare systems and institutional environments may be limited. Future studies may conduct cross-national comparisons to examine whether similar sustainability implementation patterns are observed in different healthcare contexts.

Third, although the mixed methods design provided complementary quantitative and qualitative perspectives, the qualitative sample size was relatively limited. In addition, although the questionnaire was theoretically developed based on five sustainability dimensions, some dimensions demonstrated empirical overlap during factor extraction. Future studies may further validate the factor structure using larger samples and confirmatory factor analysis. Broader stakeholder perspectives, including healthcare professionals, policymakers, and community representatives, should also be incorporated in future research to enrich understanding of healthcare sustainability implementation.

Finally, future longitudinal studies are warranted to examine how healthcare ESG implementation evolves over time and to explore the dynamic relationships among sustainability governance, digital transformation, organizational resilience, and healthcare social sustainability practices within post-pandemic healthcare environments.

## 6. Conclusions

This study investigated the factors associated with ESG implementation and healthcare social sustainability practices among Taiwanese hospitals in the post-pandemic era using a mixed-methods approach integrating quantitative survey analysis and qualitative interviews.

The findings demonstrated that ESG sustainability support, operational sustainability, and sustainability management capability significantly influenced hospitals’ healthcare social sustainability practices. Among these factors, ESG sustainability support demonstrated the strongest positive influence, followed by operational sustainability and sustainability management capability. In contrast, organizational resource pressure did not demonstrate a statistically significant negative effect.

The qualitative findings further revealed that ESG implementation within Taiwanese hospitals increasingly involves operational integration, digital healthcare support, sustainability-oriented management systems, and healthcare social sustainability practices. Participants consistently emphasized the importance of leadership support, operational coordination, digital healthcare transformation, and community health promotion activities in facilitating long-term healthcare sustainability implementation.

Overall, the findings suggest that healthcare ESG implementation increasingly functions as an integrated healthcare sustainability strategy involving operational sustainability, digital healthcare transformation, healthcare accessibility, and social responsibility practices within post-pandemic healthcare environments.

The findings of this study contribute to the growing healthcare sustainability literature by providing empirical evidence regarding the determinants of ESG implementation within Taiwanese healthcare institutions. In addition, the study highlights the importance of sustainability support systems, operational sustainability capability, and digital healthcare support for strengthening long-term healthcare social sustainability practices.

Healthcare institutions should strengthen ESG sustainability support systems, operational sustainability strategies, and sustainability-oriented management practices to enhance long-term healthcare sustainability and organizational adaptability within post-pandemic healthcare environments.

## Figures and Tables

**Table 1:** Participant Characteristics (*N* = 135).

Variable	Category	*n*	%
Gender	Male	43	31.9
	Female	92	68.1
Age	≤30 years	18	13.3
	31–40 years	39	28.9
	41–50 years	46	34.1
	≥51 years	32	23.7
Hospital Type	Medical center	28	20.7
	Regional hospital	74	54.8
	District hospital	33	24.5
Position	Administrative manager	29	21.5
	Healthcare professional	81	60
	ESG/sustainability personnel	25	18.5
ESG-related Experience	Yes	88	65.2
	No	47	34.8

**Table 2:** Characteristics of Interview Participants.

Participant	Position	Institutional Type	Interview Duration
P1	Senior hospital administrator	Regional hospital	65 min
P2	ESG/sustainability management personnel	Public hospital	52 min
P3	Senior healthcare executive	Faith-based hospital	78 min

**Table 3:** Reliability Analysis.

Construct	Cronbach’s α
ESG Sustainability Support	0.957
Operational Sustainability	0.959
Organizational Resource Pressure	0.826
Sustainability Management Capability	0.961
Healthcare Social Sustainability Practices	0.959

**Table 4:** KMO and Bartlett’s Test.

Test	Value
Kaiser–Meyer–Olkin measure of sampling adequacy	0.875
Bartlett’s Test of Sphericity, χ^2^	8870.45
df	1485
*p*-value	<0.001

**Table 5:** Exploratory Factor Analysis Results.

Factor	Representative Variables	Factor Loading Range	Eigenvalue	Variance Explained (%)
ESG Sustainability Support	ESG support systems, environmental sustainability initiatives, sustainability implementation mechanisms	0.701–0.892	9.842	31.26
Operational Sustainability	Workflow sustainability, operational efficiency, digital healthcare management	0.684–0.881	5.214	18.47
Sustainability Management Capability	Leadership support, sustainability coordination, implementation support systems	0.655–0.873	3.776	12.95
Healthcare Social Sustainability Practices	Community engagement, healthcare accessibility, social responsibility practices	0.692–0.901	2.948	10.63

Total variance explained 73.31%.

**Table 6:** Pearson Correlation Analysis.

Variable	1	2	3	4	5
1. ESG Sustainability Support	1				
2. Operational Sustainability	0.684 ***	1			
3. Organizational Resource Pressure	−0.182	−0.154	1		
4. Sustainability Management Capability	0.721 ***	0.663 ***	−0.118	1	
5. Healthcare Social Sustainability Practices	0.742 ***	0.658 ***	−0.167	0.694 ***	1

Note: *** *p* < 0.001.

**Table 7:** Multiple Regression Analysis Predicting Healthcare Social Sustainability Practices.

Predictor Variable	Standardized β	*p*-Value
ESG Sustainability Support	0.481	<0.001
Operational Sustainability	0.276	0.001
Organizational Resource Pressure	−0.083	0.157
Sustainability Management Capability	0.214	0.019

Model statistics: F = 42.318, *p* < 0.001; Adjusted R^2^ = 0.684; Durbin–Watson = 1.964.

## Data Availability

The data presented in this study are available from the corresponding author upon reasonable request. The data are not publicly available because they contain information that could compromise participant confidentiality.

## Supplementary Materials

The following supporting information can be downloaded at https://www.mdpi.com/article/10.3390/healthcare14131935/s1, Supplementary File S1. Questionnaire Items.
