# Supplementary material for: Determinants of ESG Implementation and Social Sustainability Practices in Taiwanese Hospitals: A Mixed Methods Study
Source: Healthcare (Basel). 2026 Jul 1;14(13):1935. doi: 10.3390/healthcare14131935 (PMC13362487; doi:10.3390/healthcare14131935)
Supplement: Supplementary file 1 [file healthcare-14-01935-s001.zip › healthcare-4363455-supplementary.pdf]

Supplementary File S1. Questionnaire Items

Response Scale

Unless otherwise specified, all items were measured using a five-point Likert scale ranging from 1 = Strongly Disagree to 5 = Strongly Agree.

Section 1. Environmental Sustainability and Institutional Support

1. Our institution implements carbon reduction initiatives (e.g., green energy use).
2. Our institution adopts energy-efficiency measures (e.g., energy-saving equipment).
3. Our institution reduces medical waste through optimized waste management.
4. Our institution reduces the use of disposable medical supplies.
5. Our institution promotes water conservation measures.
6. Our institution adopts green procurement practices.
7. Our institution has implemented an environmental management system (e.g., ISO 14001).
8. Our institution collaborates with government agencies or NGOs on environmental initiatives.
9. Our institution promotes environmental awareness among employees and patients.
10. Government policies provide sufficient support for sustainability implementation.
11. Government regulations clearly guide hospitals in implementing ESG and SDG initiatives.
12. Accreditation and regulatory requirements influence our institution’s ESG/SDG priorities.

Section 2. Operational Sustainability

1. Technological innovation improves our institution’s operational efficiency.
2. Professional workforce development improves operational efficiency.
3. Digital transformation improves operational efficiency.
4. Financial resources improve operational efficiency.
5. Internal management capability improves operational efficiency.

Section 3. Financial Pressure

1. Rising labor costs have increased financial pressure during the past three years.
2. Insufficient revenue sources have increased financial pressure during the past three years.
3. Cash-flow shortages have negatively affected operations during the past three years.
4. Reduced government subsidies have increased financial pressure during the past three years.

Section 4. Sustainability Management Capability

1. Our institution optimizes supply chain management to reduce costs.
2. Our institution reduces energy and water waste to improve sustainability performance.

3. Our institution simplifies operational processes to reduce duplication and waste.
4. Our institution controls unnecessary expenditures.
5. Our institution adopts digital transformation initiatives to improve efficiency.
6. Our institution develops alternative revenue sources to support sustainable operations.

Section 5. Healthcare Social Sustainability Practices

1. Our institution provides free community health screening programs.
2. Our institution offers financial assistance for vulnerable populations.
3. Our institution provides specialized healthcare services for children and older adults.
4. Our institution implements chronic disease management programs.
5. Our institution participates in public health education activities.
6. Our institution participates in vaccination and disease prevention programs.
7. Our institution organizes community first-aid and health education programs.
8. Our institution participates in mental health promotion and suicide prevention activities.
9. Cross-sector collaboration facilitates value co-creation.
10. Our institution promotes employee well-being and workplace safety as part of ESG/SDG implementation.
11. Our institution regularly discloses ESG/SDG achievements to enhance public trust.
